# Behavioral Seizure in a Patient With a Cavernous Malformation Finding in CT: A Case Report

**DOI:** 10.7759/cureus.34731

**Published:** 2023-02-07

**Authors:** Dunya Alfaraj, Maram Alismail, Hadeel Almulhim, Waad Algathami, Shahad Alabdulqader, Muath Alismail

**Affiliations:** 1 Emergency Department, Imam Abdulrahman Bin Faisal University, King Fahad University Hospital, Dammam, SAU; 2 College of Medicine, Imam Abdulrahman Bin Faisal University, Dammam, SAU; 3 Emergency Medicine, King Fahad Specialist Hospital, Dammam, SAU

**Keywords:** parietal cavernous malformation, frontal lobe, temporal lobe, cavernous malformation, behavioral epileptic seizures

## Abstract

Behavioral epileptic seizures (BES) are a unique type of seizure that can be presented with or without the classic limb movements of epilepsy. This type of seizure is commonly associated with the frontal and temporal lobes of the brain. Symptoms consist of anxiety, smiling, crying, fear, aggression, irritability, and change in awareness or activity. We report a case of unusual seizure presentation of jerky movements followed by intense fear and crying caused by a right parietal cavernous malformation with chronic bleeding.

## Introduction

Behavioral epileptic seizures (BES) are challenging and usually a misdiagnosed type of seizure [1]. Symptoms consist of anxiety, smiling, crying, fear, aggression, irritability, and changes in awareness or activity [1]. BES were found to be associated with frontal and temporal lobe abnormalities in the brain [2]. Most behavioral seizures are overlooked because they may happen alone without an ictal epileptic seizure, occur during sleep as periods of awakening, or may be misdiagnosed as behavioral disorders, especially in pediatric-age patients with intellectual developmental issues and autistic features [1]. Fear was the most common feature observed followed by depression during the ictal stage as reported by Mula M [3]. The prevalence of BES is not reported in the current literature, however, it is reported that presenting with ictal fear has a prevalence of 15%-20% in patients with mesial temporal lobe epilepsy (MTLE), which is the most common form of focal epilepsy, and it is the source of 80% of temporal lobe epilepsy (TLE) seizures [4,5]. Additionally, a multi-center study reported that the incidence of dacrystic seizures was 0.13% in all patients admitted, those seizures are presented as paroxysmal episodes of stereotyped crying having a rare ictal phenomenon [6,7]. Rougier et al. reported that 5-10% of patients with partial seizures presented with fear and panic. On the other hand, around 1% of patients with TLE presented with depression during the ictal phase [8]. There is limited literature regarding the risk factors for behavioral seizures, but there are some studies that reported age and gender differences [9]. Negative ictal affective symptoms (weeping and fear) presented more in women compared to men [9]. Moreover, ictal affective symptoms especially fear are more likely to present in young patients compared to adults [9]. There is well-established evidence that specific brain structures are responsible for ictal anxiety symptoms [4]. In this study, we report on a patient who presented to the emergency department (ED) with an unusual presentation of seizure with postictal behavioral seizure associated with intense fear and crying, which was found to be associated with a cavernous malformation (CM) in the brain.

## Case presentation

**Figure 1:**
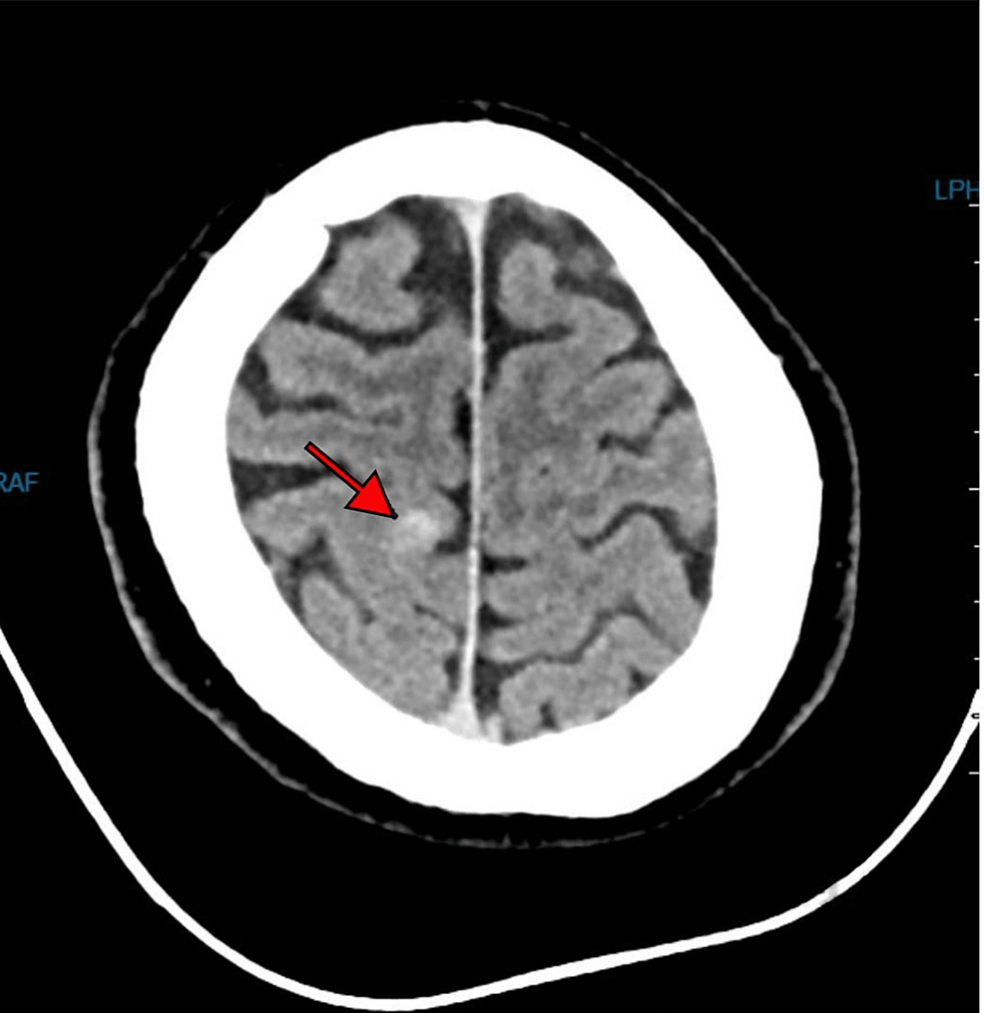
CT axial view: right parietal hemorrhagic lesion

**Figure 2:**
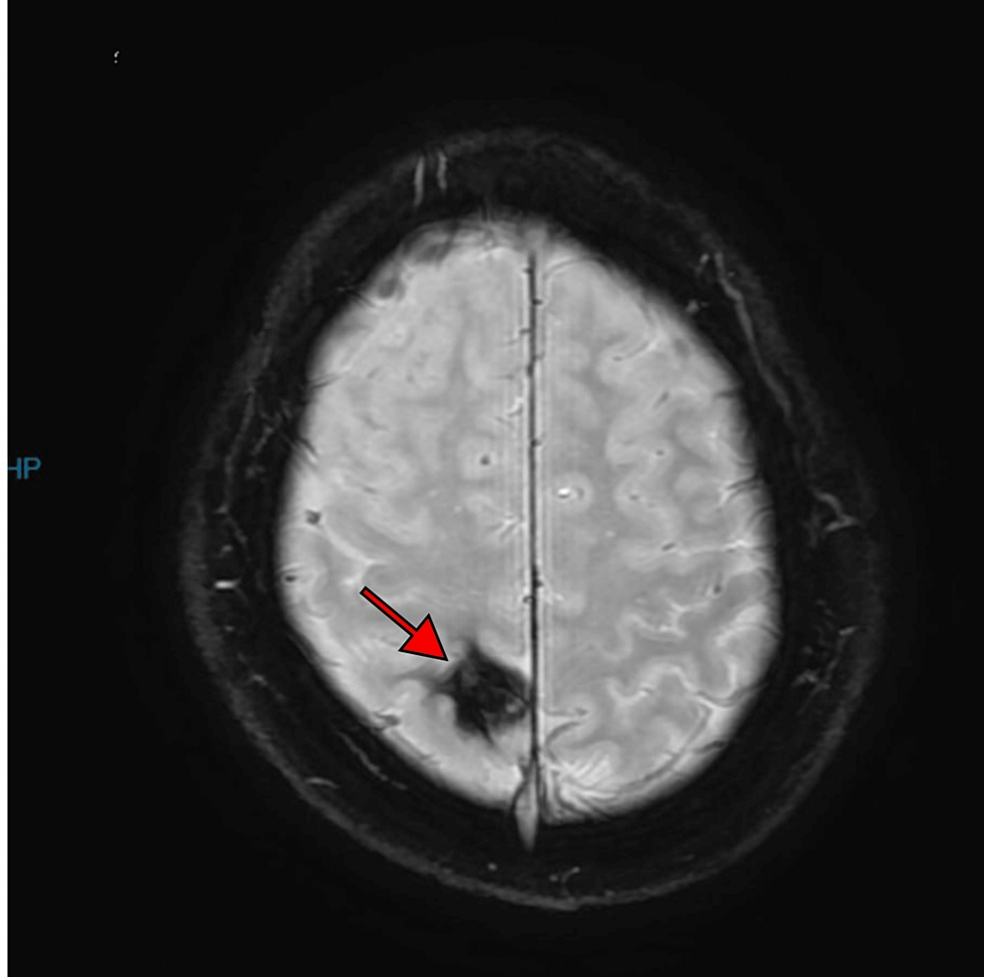
MRI axial view susceptibility-weighted image (SWI): the right parietal lesion shows a blooming artifact representing hemorrhage

A 32-year-old Saudi man, medically free, presented to King Fahad University hospital (KFUH) in Khobar, Saudi Arabia. He was admitted to the ED with a chief complaint of sudden jerky movements of upper and lower limbs that started at home, lasting for three to five minutes witnessed by his father, followed by intense fear, and crying for no obvious reason. He had no other post-ictal symptoms associated with the seizure other than the behavioral symptoms mentioned. There was no history of infection or head trauma. Neurological assessment was otherwise normal, along with unremarkable laboratory findings. EEG was done and showed no epileptiform discharges. CT scan showed a right high cortical parietal intra-axial hyperdense lesion with no edema, representing a hemorrhagic lesion (Figure 1). Therefore, MRI was done, and it showed a right parietal lesion with a blooming artifact indicating hemorrhage (Figure 2). The clinical presentation in correlation with the MRI results was highly suggestive of cavernous malformations. The patient was prescribed levetiracetam (Keppra) to manage his seizure symptoms at a loading dose of 3000 mg per oral, followed by 500 mg per oral twice a day. In addition to medication, he was advised to undergo annual MRI imaging. One month after the initial presentation, he returned to the ED after experiencing a similar episode of seizure-like activity associated with nausea and followed by intense fear and crying. Because of ongoing depressive symptoms, he was referred to a psychiatrist who eventually diagnosed him with depressive disorder secondary to his seizure condition. He was prescribed amitriptyline 10 mg per oral daily and escitalopram 10 mg per oral daily.

For the subsequent two years, whilst on medication, the patient remained free from seizure symptoms. He was controlled on levetiracetam (Keppra) 500 mg twice a day. MRI imaging was repeated twice (once a year) in this two-year period with no significant interval change regarding the previously seen right superior parietal lobule cavernoma, which shows a stable size and signal intensity indicating a stable right parietal cavernoma. During this period of time, he had frequent follow-ups in the psychiatry clinic, showing significant improvement regarding depressive symptoms.

## Discussion

CMs are low-flow vascular malformations that can be found in 0.4-0.8% of the general population [10]. They most commonly occur in the brain and, to a lesser extent, the spinal cord [10]. They are characterized by dynamic changes, including bleeding, enlargement, and shrinkage [10]. About 40% of patients with CMs remain asymptomatic, whereas some may complain of seizures (23-50%), headache (6-52%), focal neurological deficits (20-45%), and intracranial hemorrhage (9-56%) [10]. The pathogenesis of CMs is not fully understood but genetic causes have been identified recently [11]. Most CM cases are sporadic, whereas about 20% of them are familial, having autosomal dominant inheritance patterns [11]. The genes that have been found to be associated with CM are CCM1, CCM2, and CCM3 genes [11]. Our patient presented with BES caused by CM with chronic bleeding.

BES is a rarely reported type of seizure with atypical behavioral disturbances of anxiety, smiling, crying, fear, aggression, irritability, and changes in awareness or activity [1]. Fohlen et al. reported BES in the form of episodes of vigilance, decreased psychomotor activity, crying, smiling, dis-coordinated movements of the body, and complex motor automatisms in eight children [1]. Similarly, Bonini et al. reported 32 patients whose seizure episodes involved tonic contractions of the arms, random leg movements, screaming, crying, and giggling [12]. Moreover, BES could present as episodes of severe aggression that could be harmful to the patient and their surroundings [13]. In this case, the patient presented with behavioral seizures in the form of fear and crying.

It was found that patients who present with BES have brain abnormalities that are more commonly located in the frontal lobe [1]. However, BES is not limited to the frontal lobe and can be associated with the temporal lobe [2]. A study to characterize frontal lobe epilepsy (FLE) divided patients into groups according to the seizure manifestations and anatomical lesions [12]. The fourth group, in which the symptoms seemed closest to those of our patient, showed involvement of regions in the orbital and medial-prefrontal network propagating to the amygdala and anterior temporal regions [12]. This group typically exhibits behaviors of fear such as screaming or swearing with a fearful expression along with autonomic signs [12].

Cortical dysplasia in the frontal region was one of the common pathologies causing BES [1,12]. Other pathologies include diffuse atrophy, FLE, and Lennox-Gastaut syndrome, which is a severe childhood epilepsy syndrome that manifests as mixed seizures, intellectual disability, and generalized slow (<3Hz) spike-wave discharges on EEG [12,14]. In one severe case of BES, the MRI showed a right inferior frontal gyrus hyperintense lesion. Neuroimaging finding in patients with dacrystic seizures were all abnormal and included hypothalamic hamartomas, temporal sclerosis, and glioblastoma [6]. In this case, the patient's presentation was attributed to cavernous malformation in the right parietal area as shown in MRI (Figure 2).

According to Sumer et al., the EEG findings for patients who presented with aggression as BES were unremarkable, except for one occasion that showed postictal periodic lateralized epileptiform discharges (PLED) in the right frontoparietal region [13]. Similarly, the EEG in the presented case showed no epileptiform discharges. Other studies reported that behavioral symptoms were associated with epileptiform discharge in the anterior part of the brain [1]. Moreover, it was reported that patients with cortical lesions had more frequent ictal EEG discharges in the temporal region [6].

The treatment of seizure symptoms caused by CMs depends on the location of the lesion, whether it is located superficially or deep in the brain stem [10]. However, to control seizure symptoms, treatment should be started conservatively with the use of antiepileptic drugs [10]. For those patients who present with superficial CMs or intractable seizures, surgical intervention is the treatment of choice [10]. Fohlen et al. found that resecting the affected brain segments leads to improvement in intellectual ability, sleep quality, attention, and concentration as well as fewer seizure episodes [1]. While the best treatment for symptomatic deep-seated lesions is controversial [10]. Surgical resection is a treatment option but has considerable risks of morbidity and mortality [10]. On the other hand, radiosurgery is considered an alternative option but still has some risks such as permeant neurological deficits [10]. In this case, the patient showed a good response to levetiracetam.

Depression is a common complication in patients with epileptic disorders [15]. In 2017, a case report was published of a 66-year-old female with a left frontal lobe cavernous malformation associated with a long history of headaches and epilepsy [16]. Her epilepsy was of partial and generalized types seizures, manifesting as jerky movements of limbs and face accompanied by dizziness and confusion [16]. She was treated with multiple antiepileptic drugs, including carbamazepine, phenobarbital, lamotrigine, and levetiracetam [16], in addition to daily flunarizine to relieve her headaches [16]. Later in the course of the disease, she developed a psychiatric syndrome called pseudo-depression syndrome presented with apathy, loss of initiative, libido, and mood changes eventually causing depression [16]. Paroxetine was administered for her depression with close monitoring of her condition improvement [16]. In the presented case, the patient was diagnosed with depressive disorder secondary to epilepsy and was prescribed amitriptyline and escitalopram to control his depressive symptoms.

The presented case is considered the second reported case of BES associated with cavernous malformation and the first case reported (up to our knowledge) with BES secondary to a right parietal cavernous lesion. A similar case report was reported by Denton et al. of a patient who presented with drug-resistant epilepsy with two types of seizures [7]. One of the types included was sudden hypermotor activity associated with ictal and postictal aggression, and postictal confusion, which happened without warning or awareness [17]. It happens once a month since the age of 13 years old [16]. The patient had a history of ADHD and past drug abuse [17]. He was diagnosed at the age of 29 years old after presenting to the ED with a tonic-clonic seizure [17]. The brain MRI showed a lesion in the left temporal region, specifically over the left cingulate area that is suggestive of cavernous vascular malformation [17]. A video EEG recorded an episode and showed bifrontal spikes in Fp1, Fp2, F7, and Sp1 [17]. The patient was given lamotrigine 100 mg per oral twice a day and clobazam 10 mg per oral twice a day. He later underwent cavernoma resection surgery, which resulted in one year free of seizures [17].

## Conclusions

Cavernous malformations can cause convulsions or cerebral bleeding even though they are usually asymptomatic. We report the first published case of a behavioral seizure in the form of fear and crying associated with a cavernous malformation. Anti-epileptic medication is often used as the first line of treatment for seizure control, with surgical resection being superior for reducing seizure episodes and enhancing cognitive function. It is important to emphasize that people with BES or symptomatic cavernous malformations may develop psychological symptoms, which should be closely treated and monitored.
