# Correction: Behavioral Seizure in a Patient With a Cavernous Malformation Finding in CT: A Case Report

**DOI:** 10.7759/cureus.c104

**Published:** 2023-02-28

**Authors:** Dunya Alfaraj, Maram Alismail, Hadeel Almulhim, Waad Algathami, Shahad Alabdulqader, Muath Alismail

**Affiliations:** 1 Emergency Department, Imam Abdulrahman Bin Faisal University, King Fahad University Hospital, Dammam, SAU; 2 College of Medicine, Imam Abdulrahman Bin Faisal University, Dammam, SAU; 3 Emergency Medicine, King Fahad Specialist Hospital, Dammam, SAU

This article has been corrected to change the affiliations of the following authors:

- Maram Alismail
- Hadeel Almulhim
- Waad Algathami
- Shahad Alabdulqader

The affiliations of these four authors were incorrectly submitted as 'Medicine, Imam Abdulrahman Bin Faisal University, Dammam, SAU' however, the University requires that they be changed to ''College of Medicine, Imam Abdulrahman Bin Faisal University, Dammam, SAU'. The authors deeply regret this error.

